# Visual contrast sensitivity is associated with community structure integrity in cognitively unimpaired older adults: the Brain Networks and Mobility (B-NET) Study

**DOI:** 10.1016/j.nbas.2024.100122

**Published:** 2024-07-24

**Authors:** Alexis D. Tanase, Haiying Chen, Michael E. Miller, Christina E. Hugenschmidt, Jeff D. Williamson, Stephen B. Kritchevsky, Paul J. Laurienti, Atalie C. Thompson

**Affiliations:** a Wake Forest University School of Medicine, Department of Radiology, Winston-Salem, NC, USA; b Wake Forest University School of Medicine, Department of Biostatistics, Winston-Salem, NC, USA; c Wake Forest University School of Medicine, Division of Public Health Sciences, Winston-Salem, NC, USA; d Wake Forest University School of Medicine, Department of Gerontology and Geriatric Medicine, Winston-Salem, NC, USA; e Wake Forest University School of Medicine, Department of Surgical Ophthalmology, Winston-Salem, NC, USA

**Keywords:** Contrast sensitivity, Brain networks, Functional MRI

## Abstract

Older adults with impairment in contrast sensitivity (CS), the ability to visually perceive differences in light and dark, are more likely to demonstrate limitations in mobility function, but the mechanisms underlying this relationship are poorly understood. We sought to determine if functional brain networks important to visual processing and mobility may help elucidate possible neural correlates of this relationship. This cross-sectional analysis utilized functional MRI both at rest and during a motor imagery (MI) task in 192 community-dwelling, cognitively-unimpaired older adults ≥ 70 years of age from the Brain Networks and Mobility study (B-NET). Brain networks were partitioned into network communities, groups of regions that are more interconnected with each other than the rest of the brain, the spatial consistency of the communities for multiple brain subnetworks was assessed. Lower baseline binocular CS was significantly associated with degraded sensorimotor network (SMN) community structure at rest. During the MI task, lower binocular CS was significantly associated with degraded community structure in both the visual (VN) and default mode network (DMN). These findings may suggest shared neural pathways for visual and mobility dysfunction that could be targeted in future studies.

## Introduction

As individuals age, it is believed that vision becomes progressively more integral to their mobility, and older adults with visual impairment are known to be more prone to mobility dysfunction [1]. Maintaining adequate mobility is a critical component of healthy aging that can promote independence and a higher quality of life [2]. Older adults with lower mobility function are at greater risk of falls [3], fractures and mortality [4]. Population-based studies have also suggested that older adults with poor vision are more likely to experience decrements in gait and balance [5], [6], [7], [8], and are also at increased risk of falls [9], [10] and mortality [4]. Despite this connection, mechanisms connecting specific aspects of age-related visual and mobility dysfunction have not been well described.

Recent work has suggested that cognitive impairment may influence visual somatosensory integration and mobility, potentially mediating this relationship [11]. However, age-related changes in vision and mobility are also common in older adults without mild cognitive impairment or dementia [11], [12], [13]. We have reported that even small decrements in contrast sensitivity (CS) are associated with worse performance on the expanded short physical performance battery (eSPPB) among cognitively unimpaired older adults with good visual acuity in the Brain Networks and Mobility Study (B-NET) [6], [14]. We have also observed that impairment in CS may be more central to gait and balance dysfunction than discrimination of high contrast visual acuity [6], [7]. CS is the ability to perceive differences in light and dark and is important to spatial relationships and pattern recognition. The majority of the visual world is perceived in lower shades of contrast, which may explain its relative importance to mobility compared to high contrast acuity testing. Multiple age-related eye diseases are associated with impaired CS which can occur upstream of visual acuity deficits [15], but declines in CS are also observed in normal aging [16]. Together, these findings may suggest that mild CS deficits could be predictive of physical dysfunction even in older adults without cognitive impairment or acuity loss.

To more fully understand how disrupted CS may result in altered mobility dysfunction, it is essential to begin to determine the neural correlates of CS. Such neural mappings may help to elucidate novel targets upstream of disability. Our group has previously demonstrated that older adults with worse SPPB or worse eSPPB scores have degraded sensory motor cortex community structure using brain networks generated with functional magnetic resonance imaging (fMRI) data [17], [18]. Since visual and physical dysfunction are often comorbid, we hypothesized that there could be corresponding functional changes in regions of brain connectivity important to visual processing and mobility. In this analysis, we used data from the baseline visit of the B-NET study to examine whether CS was associated with community structure in subnetworks covering the entire brain. A data-driven exploration was used to identify all potential neural mechanisms that may underlie the relationships between CS and physical function. These analyses were performed for networks recorded while participants were at rest and during a motor imagery (MI) task.

## Methods

### The B-NET study

B-NET (NCT03430427) is a longitudinal, observational study of community-dwelling older adults aged 70 and older recruited from Forsyth County, North Carolina, and the surrounding areas. The study was conducted in accordance with the Declaration of Helsinki and approved by the Institutional Review Board of the Wake Forest School of Medicine (IRB protocol #IRB00046460; approval date: 08/27/2020). All participants provided written informed consent for the B-NET study.

The B-NET study inclusion and exclusion criteria have been described in detail previously [6]. In brief, the main exclusion criteria for screened participants included being a single or double amputee, having severe musculoskeletal implants that could impede functional testing dependence on a walker or another person to walk, recent surgery or hospitalization within the previous 6 months, serious or uncontrolled chronic diseases, hazardous alcohol use (>21 drinks per week), clinical manifestation of a neurological disease that affects mobility, history of traumatic brain injury with residual deficits, history of brain tumors, seizures within the last year, unwillingness or inability to undergo an MRI brain scan, plans to relocate within the next 2 years, participation in a behavioral intervention trial, or evidence of cognitive impairment. In addition, we excluded those with major uncorrected hearing or vision problems since they had to be able to complete a visual task in the scanner and follow the auditory prompts. Cognitive status was assessed with a battery of tests, including the Montreal Cognitive Assessment (MoCA), with scores below 21 being exclusionary. For participants with MoCA scores between 21 and 26, the study neuropsychologist reviewed all available cognitive test data to exclude those with suspected mild cognitive impairment (MCI) [19], [20]. The other cognitive tests included in the B-NET protocol were the Auditory Verbal Learning Test, trail making, Digit Symbol Coding, Craft Story Immediate and Delayed Recall, Word Fluency by Letter (F and L), and Category (animals, vegetables). An array of sociodemographic and clinical data were also collected at baseline, and have been described previously [18], [21].

### Visual function testing

Participants were asked to report whether they had any eye conditions such as cataracts, glaucoma, age-related macular degeneration, or retina problems, retinopathy, or other retinal diseases or changes. They were also asked to rate their current eyesight as excellent, good, fair, poor, very poor, or completely blind.

Binocular visual acuity testing was conducted using the ETDRS eye chart while wearing corrective lenses (if applicable) and standing at the standard distance of 4-m. Their visual acuity was recorded in logarithm of the minimum angle of resolution (logMAR) and Snellen visual acuity and categorized as worse than 20/40 or 20/40 or better. In B-NET, average participant binocular visual acuity was high, as good visual function was a requirement of the enrollment criteria (Table 1). Specifically, only 3 individuals (1.5 % of sample) had visual acuity scores slightly worse than 20/40 (e.g.. logMAR ∼ 0.39).

**Table 1:** Participant demographic characteristics and descriptive statistics at baseline.

	**Older Adults (N = 192)**
**Age – mean (standard deviation)**	76.4 (4.7)
**Race/Ethnicity – n (%)**	
White/Non-Hispanic	173 (89.1)
White/Hispanic Black/Non-Hispanic Asian/Non-Hispanic	2 (1.0) 18 (9.4) 1 (0.5 %)
**Sex – n (%)**	
Men	84 (43.8)
Women	108 (56.3)
**Binocular log CS – mean (SD)**	1.71 (0.14)
**Binocular logMAR Visual Acuity – mean (SD)**	0.09 (0.1)
**MoCA – mean (SD)**	25.6 (2.2)

The calculated values are means for continuous measures and counts for categorical measures. Binocular visual acuity and MOCA are set upon study enrollment restrictions, so they are not analytically incorporated as they are poor statistical variables.

CS = contrast sensitivity; logMAR = logarithm of the minimum angle of resolution; MoCA = Montreal Cognitive Assessment; eSPPB = expanded short physical performance battery.

CS was tested both binocularly and monocularly using a Pelli-Robson eye chart at a distance of 5 feet while wearing corrective lenses (if applicable). The total number of letters correctly read was recorded and converted to log contrast sensitivity for analysis [22]. Lower log contrast sensitivity indicates more dysfunction. Binocular log contrast sensitivity was selected as the independent variable of interest for these brain network analyses. In the remainder of this document, we refer to binocular log contrast sensitivity as CS for simplicity.

### fMRI protocol

**Fig. 1:**
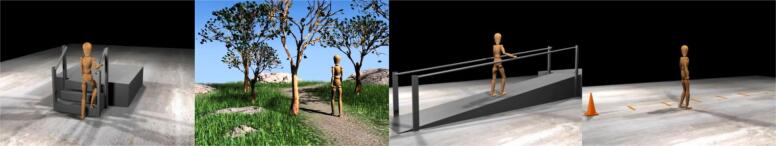
Mobility Assessment Tool–short form (MAT-sf) adaptation video clips. The MAT-sf motor imagery (MI) task shown to B-NET participants in the scanner was utilized to examine brain networks associated with MI. In total, participants are shown 10 animated video clips over the course of 4 min demonstrating common mobility movements and tasks for them to visualize. Above we show example screenshots from four of the tests.

Each B-NET participant underwent a resting-state scan and two MI tasks using video animations. The resting scan took place first, while the order of the two imagery tasks was randomized among participants. The resting state scan comprised 230 images, lasting for 460 s, while each MI task consisted of 130 images, lasting for 260 s each. The BOLD scans were conducted parallel to the anterior commissure-posterior commissure (AC-PC) using multi-slice gradient-echo planar imaging (EPI) [23]. During the scans, participants were positioned supine in the MRI scanner with a clear view of a large MR-compatible monitor positioned at the head-end of the scanner, which they observed through a mirror. In the resting-state scan, a fixation cross was displayed on the monitor, whereas for the visual imagery tasks, continuous feed videos were played on the monitor. These videos were adapted from the Mobility Assessment Tool – short form (MAT-sf) [24], [25] and were played on a computer via a standard media player (Fig. 1). Two videos were created, featuring an avatar performing mobility tasks classified as “easy” and “hard” based on previous research involving older adults [24]. For the current study, only the “easy” MI task was utilized, as comparisons between rest and task conditions had been reported in a subset of the B-NET study [21]. Prior to entering the scanner, participants received instructions and engaged in a practice session involving visual imagery. They were instructed to envision themselves as the avatar and were informed that active participation and engagement in the task were crucial for the experiment's validity. Afterwards, participants viewed shortened videos and practiced engaging in the imagery. More comprehensive information about the task instructions, training, and specific videos has been previously documented [21].

### Image analyses

#### High resolution anatomical image

For the high-resolution anatomical image, structural image segmentation was carried out using Statistical Parametric Mapping version 12 (SPM12, available at https://www.fil.ion.ucl.ac.uk/spm). The segmented gray and white matter images were combined, and any voxel with a probability value greater than 0.5 was retained, creating a mask that included the brain parenchyma while excluding non-brain tissue and cerebral spinal fluid (CSF). To ensure further accuracy, the structural images were masked, visually inspected, and manually cleaned to remove any remaining extra-parenchymal tissues. This cleaning process was performed using MRIcron software (accessible at https://www.nitrc.org/projects/mricron). Two observers independently reviewed the masked images to ensure comprehensive coverage of the entire brain. The resulting masked and cleaned T1-weighted images were then spatially normalized to the Montreal Neurological Institute (MNI) template using Advanced Normalization Tools (ANTs).

#### Functional image analyses

The image distortion correction was performed using FMRIB's “Topup” Software Library (FSL, https://www.fmrib.ox.ac.uk/fsl). The initial 10 volumes of the BOLD images were excluded to enable signal normalization. Slice time correction and realignment of the functional images were carried out using SPM12. The BOLD images were aligned with the anatomical images in their original space and then transformed to MNI space using the ANTs-derived transformation. Motion correction was implemented using Power's motion scrubbing method, which removed volumes with excessive movement (>0.5 mm FD) and excessive signal change (>0.5 DV_GM_) [26]. On average, 7.9 ± 13.9 volumes were discarded for the test conditions, and 8.8 ± 12.9 volumes were discarded for the task conditions across all older adults. Lastly, the data were subjected to band-pass filtering (0.009–––0.08 Hz) to account for low-frequency drift and physiological noise. Confounding signals, including signals from white matter, gray matter, CSF, and the 6 rigid-body motion parameters generated during the realignment process, were regressed out from the filtered data.

### Brain network generation

A voxel-wise cross-correlation analysis was performed. The preprocessed time series of each voxel was correlated with every other voxel. This computation yielded a cross-correlation matrix that contained the Pearson's correlation coefficient in each cell, representing the connectivity between each pair of voxels (nodes). From this point forward, voxels will be referred to as nodes in the network. To convert the continuous correlation values into a binary representation, a threshold was applied to the matrix. This thresholding step produced the final binary adjacency matrix, denoted as Aij, which was an N x N matrix where N represents the number of network nodes (∼20,000). Values above the threshold were assigned a value of 1, indicating the presence of a connection, while values below the threshold were set to 0, indicating no connection. The threshold value (S) was determined empirically [27] and ensured that the density of connections was similar across all participants. Specifically, S was calculated using the formula S = log(N) / log(K), where K represents the average number of connections per node. In this way, the resulting networks were constructed such that the density of connections was consistent across conditions and participants.

### Community structure statistical analyses

To determine if CS was significantly associated with functional brain networks, we utilized a distance regression methodology [28]. This statistical methodology, is a regression model implemented in MATLAB (v R2021a) and was specifically developed to examine the relationships between the spatial pattern of brain network organization and continuous and/or categorical phenotypes, while accounting for potential confounding and nuisance variables [28].

A network community refers to a group of nodes that exhibit stronger connections among themselves compared to nodes outside the community [29]. In this study, the binary network for each participant was divided into distinct communities or neighborhoods, where each node was assigned to a single community. The optimization of network partitioning was achieved through modularity, or Q [29]. A dynamic Markov process [30] often referred to as “stability” was employed using default parameters to identify the network community partition that maximized the value of Q. Due to the stochastic nature of the modularity algorithm, it was executed 100 times, and the partition associated with the highest Q value was selected for each participant in the study. Consequently, the brain network of each individual was segmented into a set of categorical communities. While the communities themselves were not labeled, each node was assigned a specific community membership, and the data could be mapped back to brain space to observe the spatial distribution of the network communities.

For group analyses, the spatial alignment of the communities was compared across participants, and a statistical measure called scaled Inclusivity (SI) was computed to quantify the spatial similarity [31]. To determine the spatial similarity to a specific resting-state network, such as the visual network (VN), predefined templates were employed as a reference when calculating SI. The templates were generated based on resting-state brain network data from 22 healthy adults in a previous study [32]. These network templates have been used in multiple other studies of community structure in older adults [18], [33] and have proven to yield meaningful outcomes. A total of 8, non-overlapping, communities were defined and included the following: visual network (VN), default mode network (DMN), sensorimotor network (SMN), basal ganglia network (BGN), central executive network (CEN), dorsal attention network (DAN), frontotemporal network (FTN), salience network (SN). SI values range from 0 to 1, where a value of 1 indicates perfect spatial alignment between the template community and communities across all participants. In practice, each node is assigned a value less than 1 depending on the spatial variability across subjects [34]. Higher SI values indicate that a particular network community remains stable throughout the group and consistently occupies comparable brain regions across individuals. Conversely, lower SI values suggest greater dispersion and variability of the community across individuals. When interpreting the results, the relative magnitudes of SI scores between groups or conditions are more informative than the absolute scores. Unfortunately, it is impossible to visualize brain maps of the SI residuals from the regression since the analyses were based on distances rather than the raw variables. Thus, community structure maps were generated by averaging node-wise SI values within each condition (e.g., SMN during rest) for the upper and lower tertiles of the independent variable of interest. These images were used to visualize the direction of significant associations and were not used for any statistical analyses. All brain images were displayed using MRIcro software [35].

**Fig. 2:**
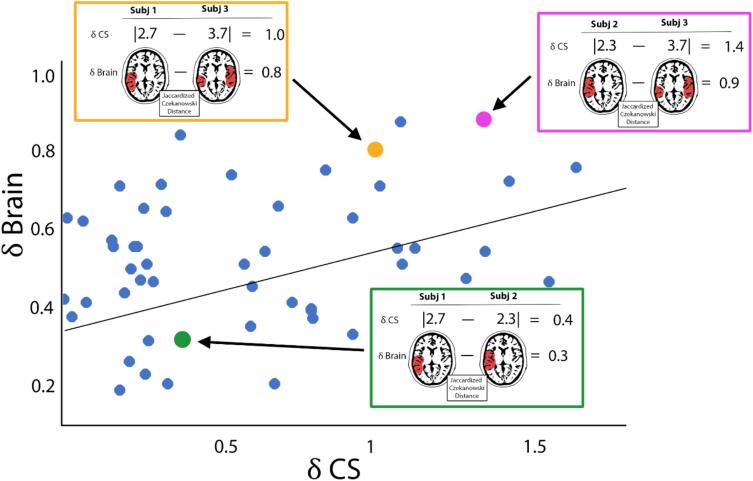
A schematic cartoon depicting the distance regression method. The brain maps and contrast sensitivity (CS) from three simulated participants from a larger group are shown above. Each participant is compared to each other participant using a distance measure. The distance (δCS) is the X value in the regression model. For the brain maps of network community structure, the weighted Jaccard distance is computed to assess spatial similarity of the two brain maps. The Jaccard distance (δBrain) is the Y variable in the regression.

To examine the associations between CS and the integrity of community structure for each brain network, separate distance regression models were constructed for both conditions (resting state and during the MI task) with the network community structure serving as the dependent variable (Fig. 2). A 3-dimensional SI brain map and the participant’s independent variables (i.e. CS) were the input for each participant. The SI maps were compared using the Jaccardized Czekanowski similarity index [36] also known as the Ružička index [37]. Note that this is a similarity index rather than a distance. To be consistent with the distances used for the independent variables, the Jaccard distance (1-Jaccard index) was used for all analyses. For the independent variable (CS), the absolute distance between participants was used. These distances were computed between every subject pair to generate a distance matrix for each variable in the model. Then, the CS distances (δCS) were regressed against the community structure distance (δBrain) using a linear statistical model with individual-level fixed effects [28]. After running the primary models for CS, adjusted models were run for each network under each condition to control for sex, as sex has been shown to be associated with SMN community structure [18]. All models were also adjusted for the number of brain volumes removed when correcting for head motion during the image preprocessing steps. Statistical significance was set at p < 0.05 for all the analyses. To account for the multiple models that were tested, an adapted false discovery rate (FDR) analysis was utilized to correct for multiple comparisons [38].

## Results

Baseline demographic data for B-NET is listed in Table 1, and the full participant characteristics can be found in the previous B-NET publication [18]. In total, 192 participants were enrolled in B-NET (mean age of 76.4 ± 4.7 years, 56.5 % female; 9.4 % black), with 98.5 % having good corrected visual acuity better than 20/40.

Table 2 delineates the associations of binocular CS with each of the eight networks covering the whole brain at rest and during the MI task while accounting for head motion and sex. Binocular CS was significantly associated with both VN (p = 0.0028) and DMN (p = 0.0016) community structure during the MI task, but not at rest (both p > 0.05). Binocular CS was significantly associated with SMN community structure at rest (p = 0.0008) but not during the MI task (p = 0.9204). These results were controlled for multiple comparisons with an adapted FDR analysis [38]. Sex and motion correction, the other variables included in our model with binocular CS, were also significant for the VN and SMN, but sex was not significantly associated with the DMN during the MI task (Table 2). None of the other brain networks exhibited associations with CS that even approached significance. Thus, the main findings reveal that CS exhibited specific associations with VN and DMN network connectivity during the MI task and SMN community structure at rest.

**Table 2:** Multivariable models of community structure differences for 8 Brain Networks at rest and during the motor imagery task.

**Network/Condition**	**Variable**	**Estimate**	**SE**	**T-score**	**p-Value**	**FDR** **p-Value**
VN/Rest	Binocular CS	−0.0012	0.0029	−0.4105	0.6815	0.9805
	Motion	0.0001	<0.0001	2.6666	0.0077	
	Sex	0.0016	0.0006	2.8931	0.0038	
VN/Task	**Binocular CS**	**0.0067**	**0.0022**	**2.9911**	**0.0028**	**0.0148**
	**Motion**	**0.0004**	**<0.0001**	**11.2087**	**<0.0001**	
	**Sex**	**0.0015**	**0.0004**	**3.3327**	**0.0009**	
DMN/Rest	Binocular CS	0.0002	0.0027	0.0688	0.9452	0.9805
	Motion	0.0004	<0.0001	10.1468	<0.0001	
	Sex	0.0031	0.0005	5.7319	<0.0001	
DMN/Task	**Binocular CS**	**0.0081**	**0.0026**	**3.1532**	**0.0016**	**0.0129**
	**Motion**	**0.0003**	**<0.0001**	**8.6057**	**<0.0001**	
	Sex	0.0006	0.0005	1.2163	0.2239	
SMN/Rest	**Binocular CS**	**0.0072**	**0.0022**	**3.3426**	**0.0008**	**0.0129**
	**Motion**	**<0.0001**	**<0.0001**	**2.2245**	**0.0261**	
	**Sex**	**0.0014**	**0.0004**	**3.3524**	**0.0008**	
SMN/Task	Binocular CS	−0.0002	0.0018	−0.0999	0.9204	0.9805
	Motion	0.0001	<0.0001	4.7271	<0.0001	
	Sex	0.0007	0.0004	2.0812	0.0374	
BGN/Rest	Binocular CS	<0.0001	0.0020	0.0325	0.9741	0.9805
	Motion	0.0005	<0.0001	15.6231	<0.0001	
	Sex	0.0008	0.0004	1.9907	0.0465	
BGN/Task	Binocular CS	−0.0002	0.0021	−0.1133	0.9098	0.9805
	Motion	0.0002	<0.0001	6.6678	<0.0001	
	Sex	0.0006	0.0004	1.4071	0.1594	
CEN/Rest	Binocular CS	−0.0001	0.0022	−0.0675	0.9462	0.9805
	Motion	0.0006	<0.0001	17.6838	<0.0001	
	Sex	0.0004	0.0004	1.0022	0.3163	
CEN/Task	Binocular CS	−0.0005	0.0019	−0.2595	0.7953	0.9805
	Motion	0.0004	<0.0001	15.0937	<0.0001	
	Sex	0.0012	0.0004	3.1201	0.0018	
DAN/Rest	Binocular CS	0.0026	0.0024	1.0753	0.2823	0.7721
	Motion	0.0001	<0.0001	3.3459	0.0008	
	Sex	0.0038	0.0005	7.9681	<0.0001	
DAN/Task	Binocular CS	0.0032	0.0021	1.5118	0.1306	0.5224
	Motion	0.0003	<0.0001	11.0300	<0.0001	
	Sex	0.0009	0.0004	2.0736	0.0381	
FTN/Rest	Binocular CS	−0.0002	0.0019	−0.1088	0.9134	0.9805
	Motion	0.0003	<0.0001	9.5023	<0.0001	
	Sex	0.0011	0.0004	2.9119	0.0036	
FTN/Task	Binocular CS	−0.0012	0.0016	−0.7326	0.4638	0.9805
	Motion	0.0002	<0.0001	7.1140	<0.0001	
	Sex	0.0003	0.0003	0.8004	0.4235	
SN/Rest	Binocular CS	0.0021	0.0020	1.0592	0.2895	0.7721
	Motion	0.0001	<0.0001	4.4308	<0.0001	
	Sex	0.0016	0.0004	4.1900	<0.0001	
SN/Task	Binocular CS	<0.0001	0.0014	0.0245	0.9805	0.9805
	Motion	<0.0001	<0.0001	3.1654	0.0016	
	Sex	0.0010	0.0003	3.7760	0.0002	

**Fig. 3:**
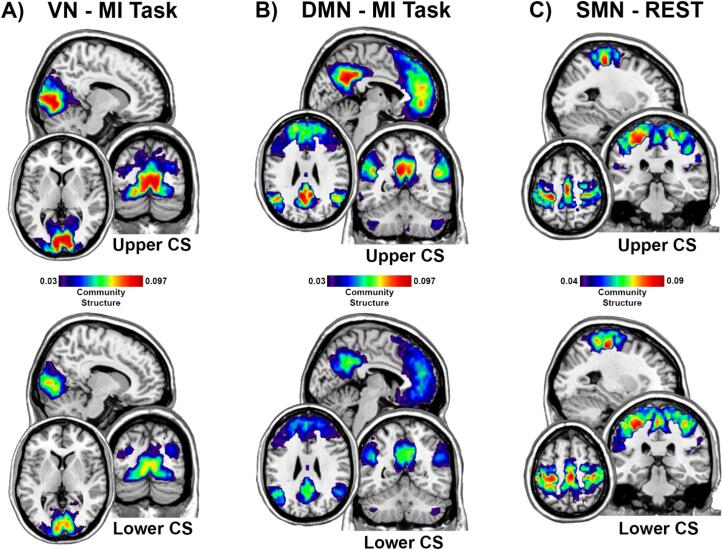
Community structure brain maps for the groups in upper and lower tertiles of contrast sensitivity (CS). A) Maps for the visual network (VN) during the MI task. B) Maps for the default mode network (DMN) during the MI task. C) Maps for the sensorimotor network (SMN) at Rest. Brain regions with hotter colors were more frequently part of the community across participants, indicating greater spatial consistency. Each image collage contains a sagittal slice (x: SMN = -1, VN = 11, DMN = -2), an axial slice (z: SMN = 55, VN = 5, DMN = -5), and a coronal slice (y: SMN = -15, VN = -74, DMN = -53).

The following table includes the adjusted community structure statistical model results. All models included binocular contrast sensitivity (CS), motion correction, and sex as the variables. 192 older adults were included in this analysis. The underlined networks/conditions p-values were all significant (p < 0.05), and correspond to the community structure images in Fig. 3. The bolded lines indicate a variable which reached significance. SE = standard error; the motion variable reflects the number of brain volumes removed during image correction. The last column denotes the p-value after a False discovery rate (FDR) procedure was applied for multiple comparisons.

SMN = sensory motor network; VN = visual network; DMN = default mode network; BGN = basal ganglia network; CEN = central executive network; DAN = dorsal attention network; FTN = frontotemporal network; SN = salience network.

The community structure maps shown in Fig. 3 visualize the direction of the association between CS and the brain network community structure. Note that these images are for visualization purposes as the statistical analyses were performed on all participants using continuous measures not group tertiles. The brain images in Fig. 3A and 3B illustrate that individuals in the lower tertile of CS exhibited degraded VN and DMN community structure relative to the CS upper tertile, respectively. In both cases there is a very distinct difference in spatial patterns between the upper and lower visualization groups (Fig. 3A, B). Lower binocular CS was associated with lower consistency in the spatial patter of the community structure. The brain images in Fig. 3C illustrate that individuals in the lower tertile of CS exhibited degraded SMN community structure at rest relative to the CS upper tertile. When examining the upper group image from the lower, a specific pattern of organization of the SMN can be assigned to each group. Specifically, the group with higher CS displays increased connectivity in the anterior portions of the SMN that are responsible for motor functions. The lower CS group demonstrated greater connectivity posteriorly in the SMN, indicating a sensory role.

## Discussion

The current study was performed to begin the search for potential neural correlates of poor CS in cognitively unimpaired older adults. The longer term goal of this work is to identify neural mechanisms that may link poor CS to poor physical function. We found that CS was associated with degraded VN and DMN network connectivity during a MI task. Lower CS was associated with altered SMN community structure at rest. However, this association was more complicated than those found for VN and DMN. This work suggests that even subtle deficits in CS in older adults without other significant visual or cognitive impairments have functional neural correlates in brain regions important to both visual and somatosensory processing.

CS has been associated with the primary visual cortex (V1) and structural components of the dorsal stream in earlier literature [39]. In the two-streams hypothesis, the dorsal stream pathway describes how visual information travels from V1 to the parietal lobe, and it is thought to be involved in processing spatial information [40]. While few studies have investigated CS in fMRI data, one suggested that fMRI volume of activation was associated with CS but not visual acuity [41]. Another demonstrated a positive correlation between CS and size of V1 on fMRI, suggesting a role in cortical magnification, which is the amount of V1 surface area dedicated to processing a fixed spatial extent on the retina [42]. We analyzed the VN, which broadly encompasses V1-V3, in order to determine if lower CS was related to degraded community structure among older adults. As hypothesized, older adults with worse CS demonstrated deteriorated VN community structure. Since the older adults in B-NET were relatively healthy, cognitively unimpaired and free from multiple significant comorbidities including significant visual issues, these findings could imply that CS is related to visual processing in the context of normal aging. While the causal direction of these relationships is not known, it is possible that poor visual input not only influences mobility, but that reduced mobility also negatively feeds back on visual processing.

Our observation that participants with lower CS had more degraded DMN community structure during the MI task may indicate that the DMN is important for vision-related balance. Greater DMN connectivity has been related to postural sway in older adults with MCI who have experienced a fall, or who have low mobility scores on the Life Space Assessment [12], [13]. In general, the DMN is thought to be involved in context-guided cognition due to its association with scene and context representation [43], [44], [45] and retrieval of memories [46], [47]. Recent literature has suggested that posterior DMN regions are activated when constructing scenes or situation models [45], [48], [49], and show greater activation with naturalistic scenes than symbolic letters [50]. Importantly, when viewing scenes, activity in posterior regions of the DMN are more strongly associated with context-dependent decisions rather than context-independent decisions [50]. Thus, when participants were presented with scenes from the MAT-sf and asked to imagine themselves performing a context-specific mobility task, it is intuitive that the posterior DMN was involved. However, since older adults with poor CS likely experience more difficulty perceiving edges and depth, it is possible that this could feedback into greater imagined difficulty when deciding how to navigate an environment. The DMN findings of this analysis thus make important contributions to understanding how deteriorated CS may influence how one introspectively moves through their surroundings (Fransson, 2006). Together these data may suggest an important relationship of CS to not only sensory motor processing in the SMN, but also context-dependent decisions participants make when introspectively navigating their environment via the DMN.

We suggest that the MI task allows us to examine the integrity of the VN and DMN while participants imagined themselves ambulating through space. Though we call it a ‘motor imagery’ task, this task is highly visual as participants were watching the MI video in the scanner and we instructed them to actively engage their visual systems and imagination. The finding that poor CS was associated with lower VN and DMN community structure, particularly during the motor imagery task, suggests that the neural embodiment of actively engaging with the environment is compromised. Moreover, this logic also applies to the DMN since the DMN is known for modulating introspection and navigating one’s environment. It makes sense that the DMN was only significant during the motor imagery task because deteriorated CS may influence how one introspectively imagines themselves moving through their surroundings.

Our data suggest that CS is related to sensory motor processing in the SMN. The SMN contains the primary motor neurons that initiate movement and has a well-documented role in maintaining mobility. Reduced SMN connectivity has been associated with poor physical function, most notably gait instability [51]. At the same time, slower gait speed has been related to increased connectivity between the SMN and frontoparietal network in older adults with MCI, and such connections have been shown to decrease following an aerobic intervention that resulted in better mobility [52]. We also have demonstrated that resting state SMN community structure is degraded in older adults with worse SPPB performance [17], and more recently confirmed this association in this cognitively unimpaired B-NET cohort using the more sensitive continuously scaled eSPPB [18]. We also have shown that CS and eSPPB are significantly associated in B-NET [6]. Thus, given the known importance of eSPPB to SMN connectivity, we investigated whether impaired CS may also be related to SMN function. The observed SMN-CS associations indicated that older adults with lower CS had dysregulated SMN community structure at rest, suggesting they had impaired sensory-motor processing, which may be related to their poor mobility. These lower CS older adults with increased posterior connectivity in their SMN may exhibit this pattern via a compensatory mechanism engaging the SMN when mobility (i.e. low CS) is impaired.

It may not be intuitive why the SMN exhibited associations at rest but not during the MI task. Our recent publication on this same B-NET cohort of older adults specifically delineates the significance of the relationship between resting state and the SMN [21]. It was proposed that conducting a motor imagery task involving complex movements likely results in increased variability in the spatial patterns of network connections among somatomotor areas. This consequently diminishes the consistency of the SMN while performing a motor imagery task in comparison to its state during rest where the SMN is more integrated. Thus, the association of CS with SMN community structure was only significant at rest, which is similar to what we have demonstrated in other studies since resting state is when the SMN community structure should be more integrated when functioning well [18].

Since functional brain networks retain plasticity, future studies should consider if longitudinal changes in physical activity are related to brain network connectivity not only in areas related to mobility but also vision. Notably, recent literature has suggested that greater physical activity may be associated with a lower risk of developing a number of age-related eye diseases, such as age-related macular degeneration [53], [54], [55], [56], diabetic retinopathy [57], [58], [59], [60], and glaucoma [61]. Mechanisms could be related to higher ocular perfusion pressures [62], improved endothelial function [57], and reduced inflammatory markers in adults with greater physical activity [58]. Recent animal work in rodents has also shown that exercise improve brain-derived neurotrophic factor (BDNF) signaling which is critical to survival of retinal ganglion cells [63], [64], [65]. Activation of such exercise-induced neuroprotective pathways may reduce risk of glaucoma, which can be likened to an accelerated aging of the retinal ganglion cells. Whether increased physical activity may also improve brain networks involved in both visual processing and mobility should be considered in future work. Accelerometer data was not available in this cohort and self-reported aerobic activity was previously shown to not be associated with brain networks in this cohort [18]. There is also literature to suggest that improving CS and acuity through surgical interventions such as cataract surgery has a positive impact on recovery of not only visual function but also cognitive performance, and corresponding regions of brain structure and function important to cognition and vision [66]. Whether similar recoveries may be experienced in mobility or regions of the brain important to mobility remains to be investigated.

Limitations of the current study include the cohort’s demographic make-up which lacked diversity. More heterogeneous samples that recruit higher proportions of non-white racial minorities will be especially important to understand the generalizability of these findings and explore any differences by race that may exist. By design the B-NET cohort only included high functioning older adults without cognitive impairment or substantial acuity deficits, which facilitated this analysis uniquely focused on CS and brain function in relatively health older adults without cognitive impairment, but this also means the cohort does not reflect the general population. These were cross-sectional analyses which precludes any inference of causality. Future longitudinal data in this cohort will help to elucidate whether these relationships persist or progress over time and may provide greater evidence for the direction of these associations. The mode of assessing CS using a Pelli-Robson eye chart is less sensitive to fine changes in the contrast sensitivity function compared to newer computerized methods that map the contrast sensitivity function across the visual field [67]. This visual testing was also not performed during fMRI scanning. Future directions may consider computerized assessments that limit visual input in older adults during neuroimaging. The scan duration was also relatively brief and while there is no consensus on optimal scan length, shorter scans may be less reliable [68] and more prone to noise [69]. Another limitation of this study is that we did not perform all analyses with visual acuity given the limited range of acuities and its lack of association with mobility in this cohort. Future studies should examine how visual acuity is related to brain function in populations across a range of types of visual functions including acuity.

## Conclusions

This study has demonstrated that lower CS was associated with degraded VN and DMN network connectivity during a MI task and altered SMN community structure at rest in cognitively unimpaired older adults. All results were significant after adjusting for sex and head motion in the MRI. The results contribute to a greater understanding of the relationship between age-dependent visual impairment and brain organization. The longer term goal of the project is to link the previously observed associations between CS and mobility dysfunction by identifying potential shared neural correlates. Whether interventions to improve visual function or to improve physical activity may impact network connectivity will be important areas to consider in future work.

## CRediT authorship contribution statement

**Alexis D. Tanase:** Writing – review & editing, Writing – original draft, Visualization, Investigation, Formal analysis, Conceptualization. **Haiying Chen:** Writing – review & editing, Validation, Investigation, Data curation. **Michael E. Miller:** Writing – review & editing, Validation, Data curation, Conceptualization. **Christina E. Hugenschmidt:** Writing – review & editing, Validation, Conceptualization. **Jeff D. Williamson:** Writing – review & editing, Conceptualization. **Stephen B. Kritchevsky:** Writing – review & editing, Funding acquisition, Conceptualization. **Paul J. Laurienti:** Writing – review & editing, Writing – original draft, Validation, Supervision, Investigation, Funding acquisition, Formal analysis, Conceptualization. **Atalie C. Thompson:** Writing – review & editing, Writing – original draft, Validation, Investigation, Formal analysis, Data curation, Conceptualization.

## Declaration of competing interest

The authors declare that they have no known competing financial interests or personal relationships that could have appeared to influence the work reported in this paper.
